# An Unusual Fatal Outcome of Laparoscopic Cholecystectomy: A Case Report

**DOI:** 10.7759/cureus.34365

**Published:** 2023-01-30

**Authors:** Catarina Osório, Diogo Silva, Luís Teles, Tiago Ferreira, Mário Nora

**Affiliations:** 1 General Surgery, Centro Hospitalar de Entre Douro e Vouga, Santa Maria da Feira, PRT; 2 Intensive Care Unit, Centro Hospitalar de Entre o Douro e Vouga, Santa Maria da Feira, PRT; 3 General Surgery, Centro Hospitalar de Entre o Douro e Vouga, Santa Maria da Feira, PRT

**Keywords:** gas gangrene, intravascular hemolysis, klebsiella oxytoca, clostridium perfringens, laparoscopic cholecystectomy complication

## Abstract

*Clostridium perfringens* (*C.* *perfringens*) and *Klebsiella oxytoca* (*K. oxytoca*) are pathogenic human bacteria. *Clostridium perfringens* sepsis with intravascular hemolysis is a catastrophic process with an extremely high mortality rate (70 to 100%).

A 74-year-old male submitted to an elective laparoscopic cholecystectomy due to cholelithiasis and develops severe abdominal pain only 10 hours after being discharged from hospital. He was admitted to the emergency department with associated jaundice, fever, and hematuria. On arrival, his hemoglobin level was 9.2 g/dL but fell to 3.4g/dL within two hours. Massive intravascular hemolysis was diagnosed and a liver abscess with gas gangrene was shown in the contrast-enhanced computed tomographic. Despite proper management, a fatal outcome was unavoidable and the patient died eight ours later. Microbiological examination isolated C. *perfringens* and K. o*xytoca.*

Liver abscesses caused by *C. perfringens *and *K. oxytoca* are extremely rare complications of laparoscopic cholecystectomy. Early recognition and prompt antibiotic therapy as well as control of septic focus are essential to minimize this fatal outcome.

## Introduction

*C. perfringens* are gram-positive, anaerobic bacteria belonging to the *Clostridiaceae* family, that inhabit the human gastrointestinal and urinary tract. Usually, the bacteria gain access to the body through penetrating wounds or mucosal defects in the gastrointestinal or hepatobiliary tract [1]. *Clostridium perfringens* sepsis with intravascular hemolysis is a catastrophic process with extremely high mortality ranging between 70 and 100% [2]. Early recognition and prompt antibiotic therapy as well as control of septic focus are essential to minimize the fatal outcome associated with this entity.

*K. oxytoca* is a gram-negative aerobic bacteria, normally associated with gastrointestinal and respiratory infections acquired by contact with colonized or infected persons and objects. These infections have a common evolution to sepsis. It is commonly seen *K. oxytoca* developing resistance to penicillin and ampicillin due to the synthesis of beta-lactamases; resistant strains against colistin and carbapenems have also been documented [3,4].

The authors present the case of a 74-year-old man with massive intravascular hemolysis, liver abscess with gas gangrene, rapid deterioration, septic shock, and multi-organ dysfunction syndrome. Microbiological examination isolated* C. perfringens *and *K. oxytoca*.

## Case presentation

A 74-year-old male with hypertension, hyperlipidemia, and benign prostatic hyperplasia was submitted to an elective laparoscopic cholecystectomy in our hospital, starting at 4 p.m., due to cholelithiasis. The surgery was uneventful, apart from a small hemorrhage from the gallbladder fossa, controlled with a laparoscopic suture. The patient was discharged without incident the next morning. At 4 p.m. he started feeling unwell with nausea and progressively worsening abdominal pain. Only 10 hours later, at 2 a.m., he was admitted to the emergency department with severe abdominal pain, jaundice, fever, and hematuria. At admission, upon physical examination, the patient presented with tachycardia, blood pressure of 154/86 mmHg, and tachypnea. Initial arterial blood gas measurement showed a pH level of 7,46 and hyperlactatemia of 7 mmol/L. Laboratory tests revealed anemia with a hemoglobin level of 9.2 g/dL that fell to 3.4g/dL within two hours and signs of hemolysis, such as red cell spherocytes and occasional nucleated red cells. Other laboratory results included the following: creatinine 1.9 mg/dl, serum aspartate transaminase (ASAT) 476 U/l, serum alanine transaminase (ALAT) 457 U/l, lactate dehydrogenase (LDH) 2300 U/l, alkaline phosphatase (ALP) 256 U/l, total bilirubin 24.15 mg/dl, indirect bilirubin 18.7 mg/dl and γ-glutamyltransferase (γGT) 543 U/l (Table 1).

**Table 1:** Laboratory tests results

Variable	Reference Value	On admission	2^nd^ Set	3^rd^ Set
Hemoglobin (g/dL)	13-17	9.2	3.4	3.7
Hematocrit (%)	40-50	26	14.9	16.7
White-cell count (10^9^/Liter)	4-11	19.8	12.9	16.8
Neutrophils (10^9^/Liter)	1.8-8	14.77	7.22	8.74
Platelet count (10^9^/Liter)	150-450	187	136	82
International Normalized Ratio (INR)	1	1.7		Hemolysis
Sodium (mmol/L)	136-145	137		139
Potassium (mmol/L)	3.5-5.1	Hemolysis		Hemolysis
Urea (mg/dL)	18-55	93		102
Creatinine (mg/dL)	0.7-1.3	1.9		2.4
Total bilirrubin (mg/dL)	0.20-1.2	24.15		14.64
Direct bilirrubin (mg/dL)	0-0.5	5.48		1.9
Indirect bilirrubin (mg/dL)	0.1-0.95	18.67		12.75
Aspartate aminotransferase (U/L)	5-34	476		Hemolysis
Alanine aminotransferase (U/L)	0-55	457		Hemolysis
Alkaline phosphatase (U/L)	40-150	256		Hemolysis
γ-glutamyltransferase (U/L)	12-64	543		
Lactate Dehydrogenase (U/L)	125-220	2300		Hemolysis
C-reactive Protein (mg/L)	<5	219		130

**Figure 1:**
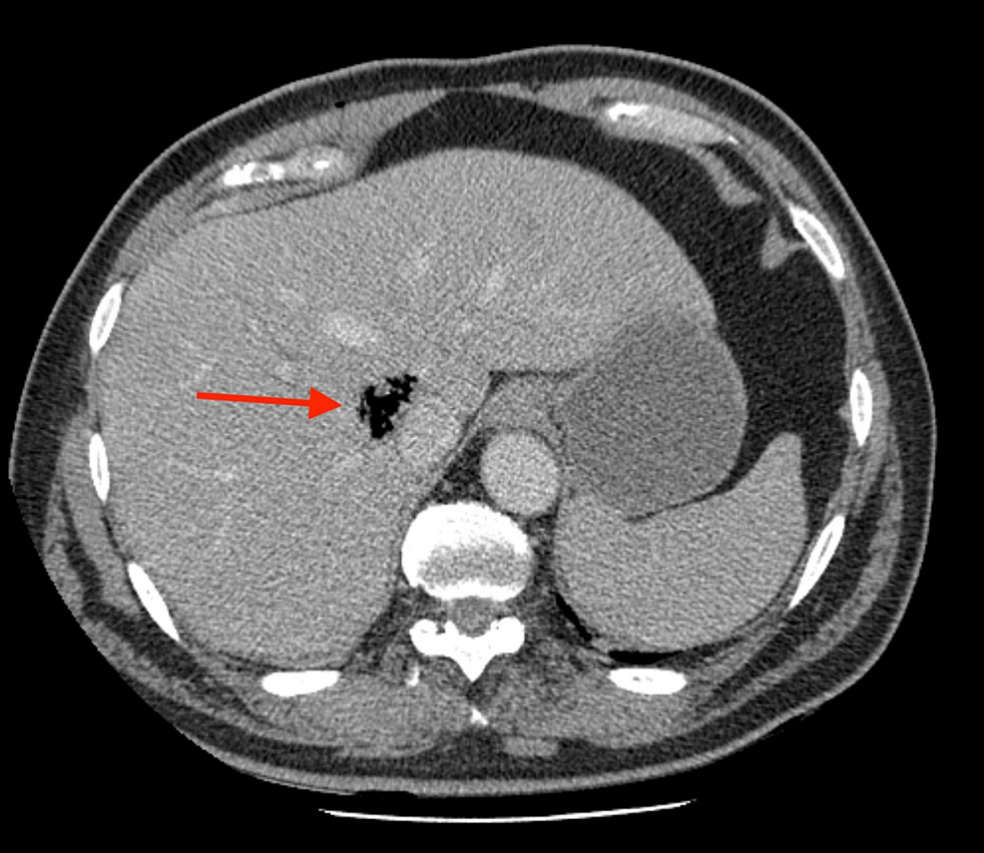
Contrast-enhanced computed tomographic (CT) of the abdomen Gas filled cavity in segment VIII of the liver near the inferior vena cava (red arrow)

After initial resuscitation, a contrast-enhanced computed tomographic (CT) of the abdomen was performed and revealed a 5cm gas-filled cavity, located in segment VIII of the liver, near the inferior vena cava. CT did not show evidence of dilated bile ducts, pneumoperitoneum, or free fluid (Figure 1).

The patient developed respiratory failure due to severe metabolic acidosis and was allocated to the emergency room. While waiting for the pending blood results and given the continuous worsening of the patient's hemodynamic status and acute hemoglobin drop, it was decided, after interdisciplinary discussion, to perform an emergent laparotomy to rule out active bleeding and to approach the gas-filled cavity identified in the CT scan. Surgery was carried out at 5 a.m. There were no signs of active or recent bleeding but drainage of the suspected abscess was not successful. The surgical team decided not to extend the surgical procedure due to the continuous hemodynamic instability of the patient during the procedure. After the surgery, the patient was admitted to the intensive care unit at 7 a.m. with inotropic support with norepinephrine and epinephrine, initiated broad-spectrum antibiotic piperacillin-tazobactam, aggressive fluid resuscitation and continuous venovenous haemofiltration was initiated for multiorgan failure, septic shock, and metabolic acidosis. Correction of coagulopathy and anemia was managed with a transfusion of fresh frozen plasma, platelets, and packed red blood cells.

The same morning, clinical pathology reviewed the blood smear and confirmed that the massive intravascular hemolysis was probably due to *Clostridium perfringens *infection. The antibiotic was switched to Meropenem plus Clyndamicin and the patient underwent percutaneous drainage of the abscess as soon as possible. Despite maximal supportive management in the intensive care unit he rapidly deteriorated and died within eight hours after admission to the ICU. Ultimately, *Klebsiella oxytoca* and *Clostridium perfringens* were isolated from blood cultures and abdominal fluid.

## Discussion

The common organisms of liver abscess formation are *Enterococcus, E. coli, Bacteroides fragilis, E. faecalis, *and* Klebsiella pneumonia*. Liver abscesses caused by *C. perfringens *and especially by *K. oxytoca *are rarely described in the literature [4]. In our case, we diagnosed a gas-forming liver abscess caused by these two bacteria.

*C. perfringens* is an anaerobic, rod-shaped, gram-positive, gas-forming, and spore-forming bacteria known to cohabit with genitourinary and gastrointestinal systems and a small percentage of gallbladders [2,4]. It is capable of producing heat-resistant spores and five types of toxins, particularly α-toxin, which damages the structural integrity of the cell, leading to tissue necrosis, gas gangrene, spherocytosis of red blood cells, and hemolysis. The isolation of the bacteria in fluid and blood cultures takes approximately 24 to 48 hours and may not be useful because culture-based antibiotic therapy delays treatment [5-7].

This bacteria is often found in the human bowel but rarely causes liver abscesses and sepsis. It is thought that *C. perfringens *sepsis presents in three different ways: contamination, clostridial crepitant cellulitis, and ultimately as gas gangrene. Massive intravascular hemolysis is only present in 7 to 15% of cases of *C. perfringens *infection [1,2]. This complication usually happens in patients with a previous history of liver cirrhosis, immunosuppressive conditions, and diabetes mellitus. Recent abdominal surgery or abortion in healthy individuals has also been reported as risk factor for infection [8,9]. The pathogenesis of *C. perfringens *sepsis after laparoscopic cholecystectomy is not yet understood. There are different theories regarding the development of bactobilia, namely entero-hepatic, ascending and hematogenous routes [2]. In our case, the symptoms began 24 hours after routine abdominal surgery.

As previously mentioned, sepsis due to* C. perfringens* has a mortality rate of approximately 80%. Therefore, early antibiotic and drainage for liver abscesses caused by *C. perfringens* and* K. oxytoca* is associated with a significant increase in the survival rate [8,9]. Regarding the choice of antibiotic therapy regime, a 2013 review of* C. perfringens *septicemia resulting in massive hemolysis revealed that penicillin in association with clindamycin had significantly reduced relative risk of death when compared with patients with different antibiotic regimes [10].

A high index of suspicion is crucial to have a proper diagnosis of* C. perfringens* following elective laparoscopic cholescystectomy. Microbiological studies can provide valuable information that can lead to the confirmation of the infection before cultures are finalized and to direct treatment strategies, as shown in this case [2]. In our patient, *Clostridium* infection was suspected before confirmatory cultures based on the clinical picture of septic shock associated with massive intravascular hemolysis and the gas-filled liver abscess. However, despite appropriate antibiotic therapy, initiated within the first hour and early percutaneous drainage of the abscess, the patient succumbed to the disease just 24 hours after admission to the emergency service and 48 hours after the elective laparoscopic cholecystectomy.

## Conclusions

In summary, massive intravascular hemolysis is a rare complication of *C. perfringens* septicaemia. This case report illustrates the importance of early recognition of this fatal complication of laparoscopic cholecystectomy.

The singularity of this case is due to *C. perfringens* septicemia resulting in massive hemolysis and also because of the microbiological detection of *K. oxytoca* in a liver abscess. To the best of our knowledge, *C. perfingens* plus* K. oxytoca* sepsis following laparoscopic cholecystectomy is extremely unusual and our case is one of the few reporting an associated gas-forming liver abscess by these two species simultaneously.
